# Efficacy and Safety of Uro-Vaxom in Urinary Tract Infection Prevention: A Systematic Literature Review

**DOI:** 10.3390/jcm14113836

**Published:** 2025-05-29

**Authors:** Silvia Volontè, Desireè De Vicari, Alice Cola, Marta Barba, Matteo Frigerio

**Affiliations:** 1 Ospedale Sant’Anna, ASST Lariana, 22100 Como, Italy; 2 Faculty of Medicine and Surgery, University of Milano Bicocca, 20126 Monza, Italy; d.devicari@campus.unimib.it; 3 Gynecology Department, Fondazione IRCCS San Gerardo dei Tintori, 20900 Monza, Italy; alice.cola1@gmail.com (A.C.); m.barba8792@gmail.com (M.B.); frigerio86@gmail.com (M.F.)

**Keywords:** urinary tract infection (UTI), *Escherichia coli*, immunotherapy, vaccine, Uro-Vaxom, pelvic floor disorders, systematic review

## Abstract

**Background/Objectives**: Urinary tract infections (UTIs) are the most common bacterial infections and one of the most common diseases worldwide. These infections induce an enormous financial and economic burden. The most frequent pathogen in UTIs is *Escherichia coli* (*E. coli*), which is responsible for over 85% of cases of cystitis and over 60% of recurrent cases. Repeated antibiotic prescriptions increase the risk of bacteria developing resistance, reducing treatment efficacy and limiting long-term therapeutic options. When traditional preventive methods fail to provide protection, other strategies may be necessary. To investigate the effectiveness of vaccination with Uro-Vaxom for the prevention of UTIs based on currently available studies. **Methods**: Systematic literature search. **Results**: The available studies focus almost exclusively on the female sex. Uro-Vaxom decreased the recurrence of UTIs, was overall well tolerated, and reduced the need for antibiotic therapies. **Conclusions**: Uro-Vaxom is a potential effective and well-tolerated option for reducing the recurrence of UTIs in patients prone to frequent infections. Nevertheless, the retrospective nature of several studies, combined with methodological limitations and variability in study design, precluded a reliable quantitative estimation of the treatment effect.

## 1. Introduction

A significant portion of the infectious process, particularly in women, is caused by urinary tract infections (UTIs), which range from asymptomatic bacteriuria to severe instances of pyelonephritis. UTIs are thought to be the most common bacterial infection and one of the most common diseases worldwide. According to the European Association of Urology (EAU), recurrent UTIs are those that occur at least twice in a 6-month period or three times in a 12-month period [1,2,3].

These infections are categorized as cystitis, prostatitis, urethritis, or pyelonephritis, based on where they first show symptoms. The most frequent of these UTIs is cystitis. When it is simple, it is a benign infection that only affects the urine and the bladder wall and does not cause fever or tissue changes. Young ladies are particularly susceptible to this disorder. Whereas an isolated episode of acute, simple cystitis is thought to be innocuous, repeated UTIs may significantly reduce women’s quality of life [4]. Moreover, these infections induce an enormous financial and economic burden on society, as recurrent UTIs can interfere with normal daily activities, reduce work productivity, and strain social and family relationships. For many patients, the constant fear of a new infection creates a state of stress and anxiety, further compromising their quality of life [5].

Therefore, the goal of treating simple cystitis should be to avoid UTI recurrences in addition to eliminating acute clinical symptoms. In certain individuals, germs can ascend to the kidneys, increasing the risk of upper urinary tract infections. These individuals include pregnant or menopausal women, the elderly, diabetics, and patients with vesicoureteral reflux (VUR) [6]. A total of 10% of women reported having a recurring UTI at some point in their life, while about 33% of women in the 20–40 age range reported having had at least one UTI treatment episode. When the urogenital microbiota and urogenital epithelium undergo negative alterations, recurrent UTIs may become more common [6,7]. A UTI during pregnancy may be a serious complication as it is associated with adverse pregnancy outcomes for both mother and child; important complications include preterm birth and small-for-gestational-age babies [8].

Elderly women frequently experience recurrent UTIs during menopause [9,10,11]. Women with bladder and bowel dysfunction are more likely to suffer from recurrent UTIs due to VUR and lower estrogen levels, which are risk factors for recurrent UTIs, particularly when there are negative alterations in the urogenital microbiome and urogenital epithelium [7].

The most frequent pathogen in UTIs, affecting both the upper and lower urinary tracts, is *Escherichia coli* (*E. coli*), which is responsible for over 85% of cases of cystitis and over 60% of recurrent cases [1,12,13]. The present pathophysiology of rUTIs can be attributed to either persistent infection or frequent recurrent ascending infection. E. coli strains cause 52–77% of rUTIs, and the microorganisms responsible for these infections are the same in the initial site of infection and in subsequent recurrences [14,15]. Certain *E. coli* serogroups have been linked to rUTIs. It is hypothesized that certain individuals may be infected with a unique strain of *E. coli* because virulence factor genes have also been independently linked to an elevated risk of persistence or relapse [15]. *E. coli* can proliferate intracellularly, and it can produce intracellular bacterial communities (IBCs), which can be challenging to identify. Antibiotic medication has the potential to keep IBCs dormant; stopping it can cause recurrence [16]. The intracellular localization and proliferation of *E. coli* within urothelial cells are major contributors to the high recurrence rates of UTIs following antibiotic treatment. Uropathogenic *E. coli* (UPEC) can evade immune clearance by forming intracellular bacterial communities (IBCs) within bladder epithelial cells. This intracellular niche not only shields the bacteria from host immune responses but also significantly impairs the efficacy of many conventional antibiotics, leading to persistent bacterial reservoirs that can subsequently cause recurrent infections. A critical limitation of antibiotic therapy is the inadequate intracellular penetration of most antimicrobial agents. Blango and Mulvey evaluated multiple antibiotic classes and observed that while many compounds effectively inhibited UPEC growth in planktonic cultures, only a limited subset, including nitrofurantoin and fluoroquinolones such as ciprofloxacin and sparfloxacin, exhibited activity against intracellular UPEC. However, even at urinary concentrations exceeding the minimum inhibitory concentration, these agents failed to fully eradicate intracellular reservoirs in in vivo models, highlighting the challenge of achieving complete bacterial clearance with antibiotics alone [17]. Another critical aspect of recurrent UTIs is their notable effect on antibiotic use. In many cases, patients rely on frequent courses of antibiotics to manage acute episodes or to prevent recurrences. While antibiotics are effective for short-term treatment, excessive and sometimes inappropriate use of these therapies significantly contributes to the well-known and growing problem of antibiotic resistance. Repeated antibiotic prescriptions increase the risk of bacteria developing resistance, reducing treatment efficacy and limiting long-term therapeutic options. This overload of prescriptions leads also to side effects such as disruption of the normal microbiota, which may, in turn, predispose patients to further infections [12,13]. In response to the developing global antibiotic resistance and the pressing need to create new and alternative strategies to fight bacterial infections, the World Health Organization created a global action plan in 2015 [18].

Preventing recurrences is a problem at present time; since immoderate prophylactic antibiotic use is extensively known to raise the risk of bacterial resistance, alternative strategies have been developed. One important strategy involves dietary and lifestyle modifications; hydration is crucial to help bacterial clearance from the urinary tract, reducing the likelihood of colonization. Maintaining good hygiene practices can help prevent the spread of bacteria from the anus to the urethra, reducing the risk of infection. Other remedies, such cranberry juice, probiotics, and chitosan have been recognized as highly effective approaches [1,19]. Lastly, D-mannose is a promising non-antibiotic prevention strategy as it prevents bacterial adhesion to the urothelium, and new research suggests that it may also act as an immune modulator [20,21]. When traditional preventive methods, such as lifestyle modifications and dietary changes, fail to provide adequate protection, other strategies may be necessary.

**Figure 1:**
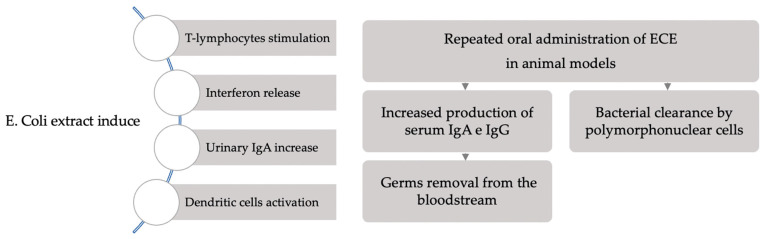
Immunological activation induced by *E. coli* extract.

In contrast to antimicrobial products, the primary objective of many alternative prophylactic approaches is not to eradicate and destroy the infectious agents but rather to safeguard the host against infection. Using the immunostimulant orally may achieve this goal by priming the patient’s mucosal immune system to respond quickly to pathogenic *E. coli* [4]. In contrast to antibiotics and natural components, vaccination represents the key point for a long-lasting protection [22,23]. Approximately forty years ago, the strategy’s proof of concept was initially presented. To stop recurrent infections in immunocompetent patients, bacterial extracts were applied empirically. Unfortunately, not knowing the precise mechanism of action made these attempts difficult. For preventing the occurrence of recurrent cystitis, the bacterial extract Uro-Vaxom (OM-89) was registered in Germany and Switzerland in 1987. Six milligrams of lyophilized bacterial lysates from eighteen strains of *E. coli*, the most common cause of UTIs, have been included in this preparation [4]. The active component of the *E. coli* extract (ECE) is the bacterial lysate, which stimulates T-lymphocytes, induces the release of interferon, raises the endogenous level of IgA in the urine, and activates dendritic cells generated from monocytes. Repeated oral administration of ECE was demonstrated in animal trials to enhance the removal of germs from the bloodstream by stimulating the production of serum IgA and IgG in mice and to initiate bacterial clearance by polymorphonuclear cells in rabbits (Figure 1) [24,25].

Over the years, several studies have investigated the role of oral immunization with Uro-Vaxom, but only few of them analyzed the clinical outcomes related to this treatment in a general population. Therefore, we aimed to perform a comprehensive descriptive review of the literature to investigate the efficacy of the treatment with Uro-Vaxom for recurrent uncomplicated UTIs.

## 2. Materials and Methods

We conducted a systematic search of the literature indexed in PubMed, Scopus, MEDLINE, and the Cochrane Library, organizing and managing references using Zotero 7. The search strategy was conducted to find all relevant abstracts regarding “Urinary tract infection”, “*Escherichia coli*”, “immunotherapy”, “vaccine”, and “Uro-Vaxom”. Boolean operators (AND, OR) were used to increase the search.

The titles and abstracts of the records that were obtained by the database searches were examined separately by two reviewers. Only articles in the English language were considered. Using the reference lists of key articles, we also manually searched for more pertinent articles. The same two reviewers separately examined the full texts of records that at least one of them recommended before determining whether to include them in the systematic review. Disagreements between reviewers were solved by consensus. Both reviewers separately extracted the data to guarantee consistency and accuracy. General exclusion criteria involved pregnancy, neurogenic urogenital disorders, vesicoureteral reflux, obstructive uropathy, urinary lithiasis, renal impairment, urologic procedures that induced UTI, severe cardiovascular disease, and hepatic insufficiency. Data were collected on patient demographic features, method of administration, type of study, year and country of publication, and period of follow-up.

A protocol registered in PROSPERO (CRD42024585075) included the study’s objectives, eligibility conditions, definitions of the results, search strategies, methods for extracting data, and evaluation of the study’s quality.

## 3. Results

**Figure 2:**
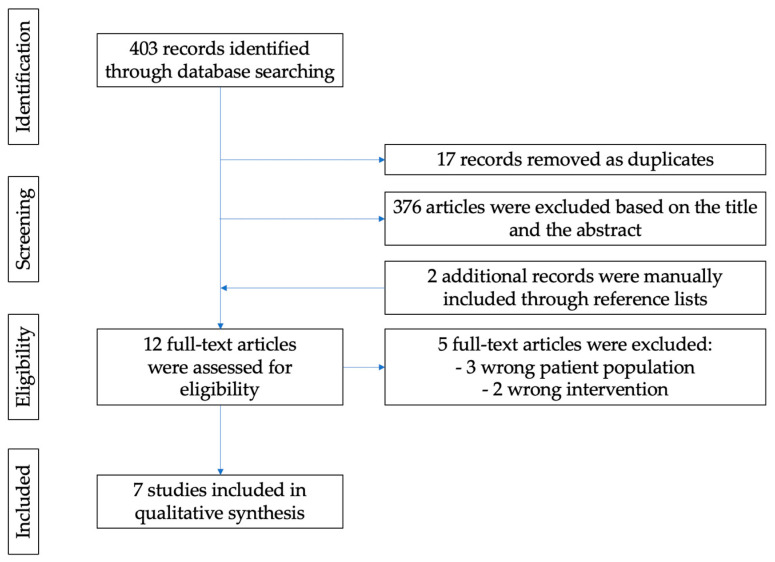
PRISMA Flow Diagram of study selection.

The electronic database search provided a total of 403 results (Figure 2). After duplicate exclusion, there were 386 records left. Of them, 376 were not relevant to the review’s purpose based on title and abstract screening. Two papers were manually added through reference list searching. Twelve studies were selected for full-text assessment, of which five were excluded for the following reasons: three papers included the wrong patient population (pediatric population or population with neurogenic bladder dysfunction), and two papers were excluded for wrong intervention (bacterial extracts different from Uro-Vaxom). A total of seven studies [2,12,26,27,28,29,30] satisfied the inclusion criteria and were integrated in the review process (Table 1). These papers included mostly randomized control studies [2,12,26,28,29], one retrospective trial [30], and one prospective trial [27]. A total of 1005 patients were included; six studies included a comparator group that was either control or placebo [2,12,26,27,28,29].

## 4. Main Findings

The principal characteristics of these studies are listed in Table 1. The included studies were appraised using the GRADE framework, which considers study design, population, intervention and comparator details, outcomes, and risk of bias. Two of the studies were robust, multicenter, double-blind randomized controlled trials (RCTs) providing high-quality evidence with low risk of bias. The remaining five studies included additional RCTs and observational or retrospective analyses. Although these additional studies were generally well conducted—with many employing double-blind methodologies—some design variations (such as non-randomized elements or retrospective data collection) introduced a slightly higher risk of bias. The studies predominantly enrolled adult patients with recurrent UTIs. Several trials focused exclusively on female populations, while others included both genders. Across studies, inclusion criteria were similar, generally requiring documented episodes of UTI (with ≥$10^{5}$ c.f.u. bacteria/mL or by at least $10^{4}$ bacteria/mL in a catheterized urine sample). The exclusion of patients with complicating factors ensured a relatively homogeneous study population.

Treatment strategy was generally homogeneous in all the analyzed studies. All the patients treated with Uro-Vaxom received one capsule daily of ECE, containing 600 mg of lyophilized immunostimulating fractions versus patients who instead received placebo in the randomized controlled trials. In almost all the records the treatment period lasted 3 months, followed by other 3 months of observation. Both Brodie and Bauer also considered the addition of a “booster” dose of Uro-Vaxom for the first 10 days of months 7, 8, and 9 [2,30]. In some of the records, follow-up was prolonged after the observational interval of 3 months right after treatment administration, depending on the study, with a maximum follow-up of 12 months. This consistency in dosing supports a direct assessment of Uro-Vaxom’s prophylactic efficacy. Primary outcomes commonly included the frequency of UTI recurrences (documented by bacteriuria thresholds) and improvements in clinical symptoms such as dysuria, urgency, and leukocyturia.

The risk of bias assessment for the included studies is summarized in Table 2. Overall, most studies demonstrated a low risk of bias across the majority of domains. RCTs consistently showed low risk of bias in key areas such as confounding, participant selection, classification of interventions, and measurement of outcomes. However, moderate risk of bias was observed in the studies by Kim and Brodie [27,30], particularly due to their non-randomized or retrospective designs, which introduced some uncertainties in confounding, participant selection, and potential deviations from intended interventions. Moreover, some studies had limitations related to sample size and loss to follow-up. Overall, while the results were consistent in favor of Uro-Vaxom, minor imprecision and heterogeneity in outcome definitions were noted. Findings across the studies were largely consistent, with each study demonstrating a favorable effect of Uro-Vaxom on reducing UTI recurrences and antibiotic use. The outcomes directly addressed the clinical question of Uro-Vaxom’s effectiveness in recurrent UTI prophylaxis, enhancing the overall directness of the evidence. Based on the GRADE assessment, the collective evidence ranges from moderate to high quality. Although the RCTs provide strong evidence, some methodological limitations in the additional studies, such as retrospective designs and modest sample sizes, warrant cautious interpretation. Nonetheless, the consistency of the positive findings supports the clinical utility of Uro-Vaxom in preventing recurrent UTIs.

The efficacy of Uro-Vaxom in reducing the recurrence of UTIs has been well documented across all the studies (Table 3). Magasi et al. reported that 67.2% of Uro-Vaxom-treated patients remained recurrence-free compared to 22.2% in the placebo group [26]. Kim et al. observed a marked reduction in the mean number of cystitis episodes, from 4.26 in the six months prior to treatment to 0.35 in the six months following treatment [27]. Similarly, Schulman et al. documented a reduction in recurrence rates, with a mean of 0.82 episodes in the Uro-Vaxom group versus 1.8 episodes in the placebo group over a 6-month period [29]. Furthermore, Bauer et al. reported a 34% relative reduction in UTI relapse rates among patients treated with Uro-Vaxom compared to placebo [2]. Additional evidence from the study by Brodie et al. confirmed a significant decrease in the mean number of UTIs, from 3.14 in the year preceding treatment to 1.53 in the year following Uro-Vaxom administration (*p* < 0.05) [30]. Tammen et al. conducted a double-blind trial with 120 patients, where Uro-Vaxom demonstrated superiority over placebo, leading to a significantly lower number of recurrences during a six-month period [28]. Long-term protective effects were also observed, with patients who had initially received Uro-Vaxom experiencing fewer recurrences even after a 5-month observation period without treatment. Similarly, Frey et al. showed that Uro-Vaxom significantly reduced bacteriuria and dysuria [12].

Secondary outcomes involved patient satisfaction and safety profiles. Tolerance to treatment was considered good in all the studies; the most frequent side effects reported included urethral symptoms, exanthema, gastrointestinal discomfort, headache, and increased blood pressure. There were no reports of anaphylaxis.

Brodie et al. evaluated overall satisfaction among patients after being treated with Uro-Vaxom, demonstrating that an over 80% positive response was provided by Uro-Vaxom responders [30].

## 5. Discussion

Recurrent UTIs significantly impact patients’ quality of life as the related debilitating symptoms, like painful urination, urgency, and increased urinary frequency, often lead to considerable physical and psychological discomfort. In addition to medical treatments, several conservative approaches can help prevent recurrent UTIs. Uro-Vaxom has shown to be effective in preventing recurrent UTIs. The present literature analysis confirms that Uro-Vaxom significantly reduces the recurrence of urinary tract infections (UTIs), with reported reductions ranging from approximately 34% to over 80% compared to placebo. In addition to this clear prophylactic effect, patients also reported improvements in subjective well-being, including decreased urinary symptoms and high treatment satisfaction. Importantly, the safety profile of Uro-Vaxom was consistently favorable across all studies, with no serious adverse events reported. The strengths of this analysis lie primarily in the quality and consistency of the included evidence. Most of the reviewed studies were randomized controlled trials (RCTs), with several employing double-blind methodologies and placebo comparators. Despite differences in sample size and follow-up duration, the direction of the results was homogeneous, consistently supporting the efficacy of Uro-Vaxom. Notably, nearly all studies shared a homogeneous treatment protocol, with Uro-Vaxom administered as a daily 600 mg oral capsule for three months, followed by an observational period—this consistency strengthens the comparability of results and reinforces the clinical applicability of the findings. This convergence of objective clinical outcomes with subjective improvements and good tolerability reinforces its role as a valuable non-antibiotic prophylactic option for recurrent UTIs. Nonetheless, several limitations must be acknowledged. First, a pooled quantitative analysis was not feasible due to heterogeneity in outcome reporting, variation in follow-up durations, and inconsistencies in how recurrence was defined and quantified across studies; not all the included studies provided sufficient numerical data to allow for meaningful aggregation or comparative analysis. The lack of a standardized effect size across trials limits the ability to present a unified estimate of Uro-Vaxom’s efficacy. Additionally, some individual studies exhibited methodological weaknesses. For instance, observational and retrospective designs inherently carry a greater risk of bias, including recall bias and lack of randomization. Small sample sizes and incomplete follow-up in certain trials also reduce internal validity. Furthermore, as shown in Table 1, the vast majority of study participants were women, and some studies included exclusively female cohorts. This gender imbalance significantly limits the generalizability of findings to male populations, for whom evidence remains limited or inconclusive.

In light of these considerations, future studies should aim to include more balanced gender representation, standardize outcome definitions, and report quantitative effect sizes transparently. Well-powered RCTs with longer follow-up periods would also strengthen the evidence base, particularly for male patients and other underrepresented subgroups.

The consistent reduction in UTI frequency, along with the decreased reliance on antibiotic therapy, supports its role as a valuable alternative to traditional prophylaxis. The EAU guidelines state that the use of immunoactive prophylaxis to reduce recurrent UTIs has a strong recommendation. As a result, it may be suggested for females with rUTIs [31]. Future research could benefit from further exploration of Uro-Vaxom’s long-term effects and its comparison with alternative prophylactic strategies such as low-dose antibiotics.

## 6. Conclusions

Uro-Vaxom has a potential therapeutic role in the prevention of recurrent UTIs. Nevertheless, the retrospective nature of several studies, combined with methodological limitations and variability in study design, precluded a reliable quantitative estimation of the treatment effect. Furthermore, the marked predominance of female participants in all included studies significantly restricts the generalizability of these findings to male patients. Future high-quality randomized controlled trials with larger, gender-balanced cohorts and standardized outcome measures are warranted to confirm and extend these results.

## Figures and Tables

**Table 1:** Characteristics of study design and population, sample size, Uro-Vaxom dosing, and follow-up.

First Author Year, Country	Study Design	Population	Mean Age	Gender Representation	Follow-Up	Dose
Bauer, HW 2005, Switzerland [2]	Multicenter, randomized double-blind controlled study	454 (232 intervention group; 222 placebo group)	41.7 ±15.3 (intervention), 39.8 ± 15.1 (placebo)	100% female	12 months	600 mg daily for 90 days or placebo then 600 mg daily for the first 10 days of months 7–9 or placebo
Kim, KS 2010, South Korea [27]	Prospective multicenter trial	34	56.4 (range 34–75)	100% female	8 months	600 mg daily for 90 days or placebo then 600 mg daily for the first 10 days of months 7–9 or placebo
Brodie, A 2020, USA [30]	Retrospective trial	79	56 (range 19–90)	95% female	12 months	600 mg daily for 90 days
Tammen, H 1990, UK [28]	Randomized controlled trial	120	51.2 ± 2.4 (intervention), 50.4 ± 2.3 (placebo)	52 women/9 men in control group vs. 51 women/8 men in placebo group	8 months	600 mg daily for 90 days or placebo
Schulman, CC 1993, USA [29]	Multicenter, randomized double-blind controlled study	142 (74 under Uro-Vaxom, 68 under placebo)	Not reported	Not specified	3 months	600 mg daily for 90 days or placebo
Frey, C 1986, Switzerland [12]	Multicenter, randomized double-blind controlled study	64 (32 under Uro-Vaxom, 32 under placebo)	Not reported (age range 22–84)	64 patients, “mostly women”	3 months	600 mg daily for 90 days or placebo
Magasi, P 1994, Switzerland [26]	Multicenter, randomized double-blind controlled study	112 (58 under Uro-Vaxom, 54 under placebo)	Not reported (age range 16–82)	48 women/10 men in control group vs. 47 women/7 men in placebo group	6 months	600 mg daily for 90 days or placebo

**Table 2:** Risk of bias summary for the included studies.

Study	Bias Due to Confounding	Bias in Selection of Participants	Bias in Classification of Interventions	Bias Due to Deviation from Intended Interventions	Bias Due to Missing Data	Bias in Measurement of Outcomes	Bias in Selection of the Reported Result
Bauer	Low	Low	Low	Low	Low	Low	Low
Kim	Moderate	Moderate	Low	Moderate	Low	Low	Moderate
Brodie	Moderate	Moderate	Low	Moderate	Moderate	Moderate	Moderate
Tammen	Low	Low	Low	Low	Moderate	Low	Low
Schulman	Low	Low	Low	Low	Low	Low	Low
Frey	Low	Low	Low	Low	Low	Low	Low
Magasi	Low	Low	Low	Low	Low	Low	Low

**Table 3:** Summary of clinical outcomes, safety, and gender distribution.

First Author	UTI Recurrence Reduction	Side Effects Reported	Patient Satisfaction
Bauer, HW	1.28 → 0.84 (34% reduction, *p* < 0.003)	Good tolerability; no serious adverse effects	Not reported
Kim, KS	Mean number of UTI: 4.26 → 0.35 (*p* < 0.001)	2 patients excluded for gastrointestinal symptoms	Not reported
Brodie, A	Mean number of UTI: 3.14 → 1.53 (*p* < 0.05)	9.4% (rash, gastrointestinal)	60.4% positive satisfaction
Tammen, H	Mean number of UTI: 3.5 → 0.82 in control group vs. 3.5 → 1.8 in placebo group	4.5% (pruritus, diarrhea, headache with flushing)	Not reported
Schulman, CC	Mean number of UTI: 1.5 placebo vs. 0.7 Uro-Vaxom (*p* < 0.0001)	2% Uro-Vaxom vs. 6% placebo (gastrointestinal, rash, vertigo, sleep disorders)	Not reported
Frey, C	Significative reduction in control group (qualitative data)	1 case of allergic exanthema	Not reported
Magasi, P	67.2% no recurrence vs. 22.2% placebo (*p* < 0.0005)	None reported	Not reported

## Data Availability

The data presented in this study are available on request from the corresponding author.
